# Influence of Pregnancy on Sexual Desire in Pregnant Women and Their Partners: Systematic Review

**DOI:** 10.3389/phrs.2023.1606308

**Published:** 2024-01-19

**Authors:** Francisco Javier Fernández-Carrasco, Cirenia Batugg-Chaves, Azahara Ruger-Navarrete, Francisco Javier Riesco-González, Rocío Palomo-Gómez, Juan Gómez-Salgado, Luciano Rodriguez Diaz, María Dolores Vázquez-Lara, Javier Fagundo-Rivera, Juana Maria Vázquez-Lara

**Affiliations:** ^1^ Nursing Department, Faculty of Health Sciences of Ceuta, University of Granada, Granada, Spain; ^2^ Nursing Department, Faculty of Nursing of Algeciras, University of Cádiz, Cádiz, Spain; ^3^ Department of Obstetrics, La Linea de la Concepción Hospital, Andusian Health Service, Seville, Spain; ^4^ Hospital Punta de Europa, Algeciras, Spain; ^5^ Department of Sociology, Social Work and Public Health, Faculty of Labour Sciences, University of Huelva, Huelva, Spain; ^6^ Escuela de Posgrado, Universidad de Especialidades Espíritu Santo, Guayaquil, Ecuador; ^7^ Health Area Campo de Gibraltar, Andusian Health Service, Seville, Spain; ^8^ Centro Universitario de Enfermería Cruz Roja, Sevilla, Spain

**Keywords:** pregnancy, sexual desire, pregnant women, sexual partners, sexual dysfunctions, public health

## Abstract

**Objectives:** Pregnancy is a stage in which different physical and psychological changes take place that can affect the sexuality of the couple. The aim of the study is to identify how the physical and psychological changes derived from pregnancy affect the sexual desire of women and men.

**Methods:** A systematic review of the literature was carried out in five databases, from which a total of 16,126 documents were obtained. After applying the PRISMA selection criteria, a total of 19 documents were selected.

**Results:** Levels of sexual desire fluctuate during pregnancy, being the second trimester of gestation the period in which desire is at its highest and in which physical limitations and emotional changes decrease. Women have lower levels of sexual desire in the first trimester, while men have the lowest levels of desire in the third trimester.

**Conclusion:** Pregnancy is a stage marked by physiological and psychological changes that modify several areas, including sexuality. Healthcare professionals should promote a healthy sexuality, avoiding the appearance of fears or sexual dysfunctions caused by the changes that occur during pregnancy.

## Introduction

Sexual and marital relationships are influenced by physiological and anatomical changes that occur during pregnancy, as well as psychological and social factors, economic conditions, religious beliefs, and gender discussions, and these vary and progress as gestation increases [1–3]. Altered body image, reduced sense of charm for the spouse, fear of injury to the foetus or fear of abortion, and early childbirth can affect women’s sexual response and ultimately the couple’s relationship [4, 5]. These factors could lead to abandonment of sexual activity and feelings of guilt regarding sexual relations during pregnancy [6]. Other couples showed a preference for sexual positions that provide for greater control over vaginal penetration and may differ from non-pregnant habits. There also appears to be greater engagement in noncoital sexual relations [3]. Some authors concluded that sexual desire could be described as a motivational state [7, 8], and pregnant women may avoid sexual intercourse or inevitably suffer from many problems in their sexual lives [1].

There is diversity in women’s and men’s sexual desire variability, with relevant practical implications in the couple sexual self-concept and sexual relationship. Women appear to show greater variability in sexual attitudes, behaviours, and attraction [9]. The first trimester is linked to a decrease in sexual relationships frequency due to nausea, sickness, breast sensitivity, and a worsening sense of wellbeing [10, 11]. Increased age is a significant risk factor for sexual distress and low sexual desire in this stage [6, 12]. In the second trimester, the frequency of sexual intercourse increases, and relates to a higher sense of security and increasing sexual interest [13, 14]. In addition, a smaller number of physical symptoms connected with pregnancy are experienced during the second trimester, improving women’s sense of wellbeing and, thus, increasing sexual desire [10, 15]. In the third trimester of pregnancy, women suffer from pain during sexual relations significantly more than in previous trimesters. Also, anatomical changes affecting the pregnant body and concerns about the health of the child influence this trimester. Thus, a great decrease in sexual interaction in this stage is associated with the pain domain [3, 10, 16, 17].

A man, even though it is not his body that has to gestate, can also experience psychological and physiological changes during pregnancy. The most extreme example is the so-called Couvade syndrome, which consists of experiencing symptoms such as vomiting, dizziness, abdominal pain or changes in appetite in a strange empathy with your partner [18].

Pregnancy can affect male sex drive. There are men who declare to have a lower sexual desire during pregnancy, and this may be due, apart from the changes that their partner is going through, to certain fears such as being able to harm the woman or the foetus during intercourse or the feeling of guilt that the pregnancy does not develop properly due to the fact of having sexual intercourse [19].

Likewise, poor relationship quality may negatively affect sexual functioning, and the link between the quality of the couple’s relationship and women’s sexuality during pregnancy has already been noted [17]. The sexuality of new parents plays an important medical and psychological role, but pregnant couples tend to avoid discussing their sexual problems [3]. Is has been assumed that women’s desire is more sensitive to the psychosocial context, and hence more variable and sensitive to major life events [9]. In this context, desire for pregnancy could be an indicator of more committed, intimate, and long-term relationships, being relevant for women’s desire in the antenatal period [20].

The aim of this review was to determine the current state of sexual desire during pregnancy by assessing the available literature to identify the most relevant physical and psychological factors that may affect couple’s sexual desire in the prenatal period.

## Methods

### Research Design

A systematic literature review was conducted to assess changes in sexual desire in pregnant women and their partners during pregnancy. The review was conducted using the systematic review format, following the criteria of the updated PRISMA 2020 guidelines (Preferred Reporting Items for Systematic Reviews and Meta-Analyses) [21]. The implemented protocol was registered in the International Prospective Register of Systematic Reviews (PROSPERO) with code CRD42023391131.

### Search Strategy

For the formulation of the research question, the PECO (Patient, Exposure, Comparison, Outcomes) format was used (Table 1).

**TABLE 1:** Research question in Patient, Exposure, Comparison, Outcomes format (Sexual desire, Spain, 2023).

Patient	Woman and sexual partner
Exposure	Physical and psychological changes during pregnancy
Comparison	No pregnancy
Outcomes	Changes in the sexual desire of the pregnant woman during each trimester of pregnancy, changes in the dyadic sexual desire of the sexual partner during each trimester of pregnancy, similarities and differences between male and female sexual desire throughout pregnancy

### Research Question

How do the physical and psychological changes derived from pregnancy affect the sexual desire of women and their sexual partners?

The following medical subject heading (MeSH) descriptors were used: sexual desire, libido, sexual dysfunctions, and pregnancy. To expand the scope of the search, free terms were added to the search using the Boolean operators AND and OR (Table 2).

**TABLE 2:** Terminology used in the search (Sexual desire, Spain, 2023).

MESH terms
Pregnant woman OR Pregnancy OR Pregnant women OR expecting mother
Sexual desire
Libido
Sexual dysfunction or Sexual dysfunctions

Table 3 shows the selection process, which was carried out between 6 and 10 May 2023 in several databases (Scopus, Pubmed, WOS, Cuiden, and Embase) using the different search strings and filtering from 2013 to February 2023.

**TABLE 3:** Document search and selection strategy (Sexual desire, Spain, 2023).

Database	Date of search	Search strategy	Documents
Scopus	6 May 2023	(sexual AND desire AND pregnancy)	92
		(libido AND pregnancy)	825
		(sexual AND dysfunction OR sexual AND dysfunctions AND pregnancy)	1,671
Pubmed	7 May 2023	(sexual desire [MeSH Terms] AND (Pregnancy [MeSH Terms])	240
		(libido [MeSH Terms] AND (Pregnancy [MeSH Terms])	240
		((sexual dysfunction[MeSH Terms]) OR (sexual dysfunctions[MeSH Terms])) AND (pregnancy[MeSH Terms])	918
WOS	12 May 2023	(TS= (sexual desire)) AND TS= (pregnancy)	2,288
		(TS= (libido)) AND TS= (pregnancy)	809
		((TS= (sexual dysfunction)) OR TS= (sexual dysfunctions)) AND TS= (pregnancy)	2,432
Embase	12 May 2023	(“sexual desire”/exp OR “sexual desire”) AND (“pregnancy”/exp OR “pregnancy”)	1,246
		(“libido”/exp OR “libido”AND (“pregnancy”/exp OR “pregnancy”)	986
		(“sexual dysfunction”/exp OR “sexual dysfunctions”) OR (“sexual dysfunctions”/exp OR “sexual dysfunction”) AND (“pregnancy”/exp OR “pregnancy”)	4,264
Cuiden	12 May 2023	(“sexual”)AND((“desire”)AND(“pregnancy”))	32
		(“libido”)AND(“pregnancy”)	3
		(“sexual”)AND((“dysfunction”)OR((“sexual")AND((“dysfunctions”)AND(“pregnancy”)))	83
Total			16,129

### Selection Criteria

The following inclusion and exclusion criteria were used for the selection of articles.

Inclusion Criteria:

• Experimental, analytical, and observational studies.
• English or Spanish language.
• Published between 2013 and 2023.
• Related to the objective of the study.
• After reading full text: articles were excluded if they were out of the scope of this review, whether they were not related to the main objective, or if data analysis was too limited or biased [22].

Exclusion Criteria:

• Bibliographic reviews, opinion articles, letters to the editor.
• Studies conducted on animals.

### Data Collection and Extraction

The search was carried out independently by two reviewers using MeSH descriptors and Boolean operators in the search strategy. The articles were selected according to the selection criteria. In case of disagreement, a third independent author, through a feedback process, made the decision.

### Methodological Quality Assessment

The assessment of methodological quality was performed independently by both reviewers using the critical appraisal tool for different studies of the Joanna Briggs Institute (JBI) at the University of Adelaide [22]. These critical assessment tools are instruments designed to help reviewers assess the methodological quality of the primary studies included in a systematic review. These tools have made it possible to standardise the review criteria, as well as to facilitate decision-making when it comes to including or excluding studies. As mentioned, JBI provides specific tools to evaluate different types of studies, such as quasi-experimental studies, observational studies, or qualitative studies, among others. This ensures that the assessment is relevant and specific to the type of study design included in the review, which contributes to the rigor and validity of systematic reviews.

In this sense, three questionnaires were used in this review, depending on the design of the selected studies. One was the eight-item JBI checklist for analytical cross-sectional studies, for which the cut-off point for eligibility was set at 6/8 by consensus of both researchers. Another one was the ten-item JBI critical appraisal checklist for qualitative research, for which the cut-off point for eligibility was agreed at 8/10. Finally, the 9-item JBI checklist for quasi-experimental studies (non-randomized experimental studies) was used, with a cut-off point of 7/9.

## Results

**FIGURE 1:**
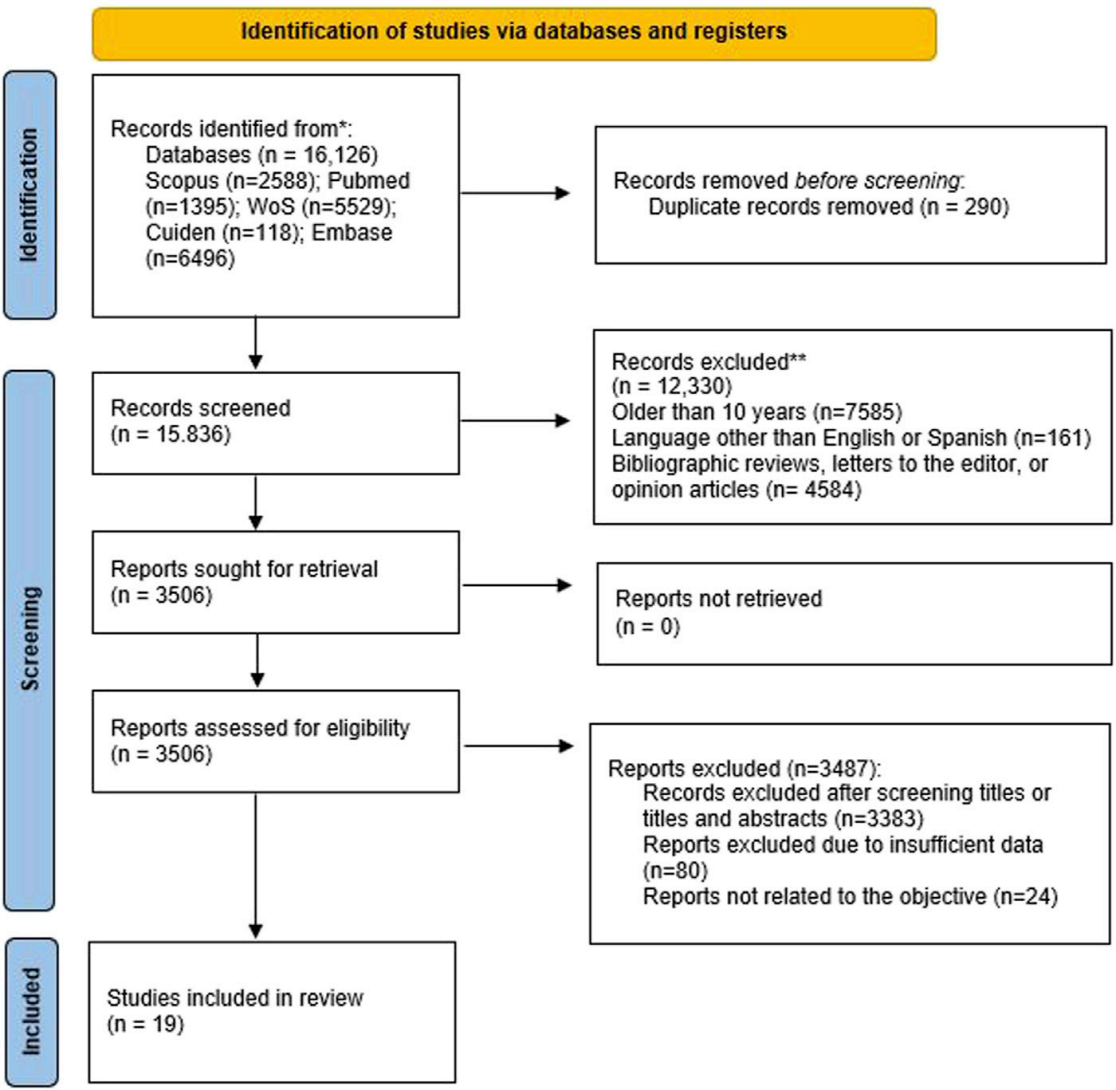
PRISMA 2020 flow diagram for new systematic reviews which included searches of databases and registers only. *Consider, if feasible to do so, reporting the number of records identified from each database or register searched (rather than the total number across all databasesiregisters). **If automation tools were used, indicate how many records were excluded by a human and how many were excluded by automation tools.

In the previously mentioned databases and using the search strings in Table 3, a total of 16,129 articles were identified. After removing duplicate articles (290), a total of 15,836 remained eligible. Then, 12,330 articles were excluded as they did not meet the inclusion criteria, leaving a total of 3,506. After reading the titles and abstracts, 3,383 other articles were excluded as they were out of the scope of this review. Subsequently, 104 articles were eliminated after reading the full text, as the data analysis was too limited (*n* = 80), or they were not related to the main objective (*n* = 24). Figure 1 shows the process followed for the identification, screening, and selection of the studies included in this review. Finally, 19 articles that assessed the changes in sexual desire in the couple during pregnancy were included in the review [1, 19, 23–39].

Of the 19 articles, there were 14 cross-sectional studies, one quasi-experimental study, and one qualitative study. Five of these articles were conducted in Turkey, five in Iran, three in Spain, one in the USA, one in Greece, one in Cuba, one in Brazil, one in Lebanon, and one in Paraguay.

The characteristics of the different studies included in this review are shown in Table 4.

**TABLE 4:** Characteristics of the studies included in the systematic review (Sexual desire, Spain, 2023).

Study	Country	Type of study	Study objective	Sample	Methods	Main findings	JBI
Saniei, 2022 [23]	Iran	Quasi-experimental study	To determine the effect of mindfulness on sexual desire and sexual satisfaction in primigravida pregnant women	72 primigravida pregnant women	FSFI (before the intervention, a week, and a month after the intervention)	Mindfulness can increase sexual satisfaction. However, sexual desire can be affected by various factors such as the cultural context, the quality of marital relationships, and the pregnancy hormonal changes that can influence the sexual response	7/9
Fernández-Sola, 2018 [24]	Spain	Qualitative study	To explore and understand the sexual experiences of expectant mothers during their pregnancy	15 expectant women	5 took part in a focus group (FG) and 10 in in-depth interviews (IDI)	Sexual intercourse during pregnancy changes in quantity and quality. Some couples become hyper-protective and due to fear of causing harm to the pregnant woman or the foetus, reduce the frequency of sexual intercourse, bearing in mind that many couples see penetration as a necessary factor for a satisfying sexual relationship. In addition, in the first trimester of pregnancy, symptoms such as nausea led to a decrease in the frequency of sexual relations, as well as in late pregnancy, when the pregnant woman is not physically well enough to have sexual relations. All this affects sexual desire, which fluctuates throughout pregnancy, being higher during the second trimester, when physical limitations are lower than in the other trimesters. That is why it is important, in order to maintain good sexual health, to adapt to the new physical limitations of pregnancy, to a new form of sex that looks beyond penetration, and to try new positions that are comfortable during this stage	9/10
García-Mazón, 2016 [25]	Spain	Cross-sectional study	To describe the changes that occur in the sexual desire of women during pregnancy	50 expectant women	23-item questionnaire	As pregnancy progresses, couples readjust their sexual and cohabitation expectations to adopt the new role of mother/father, and changes in the sexual response of the couple are observed. During pregnancy, levels of desire, excitement, and orgasm are observed to decrease as the pregnancy progresses. However, there is an increase in desire and excitement during the second trimester due to reduced discomfort and emotional changes that mark the first and third trimesters of pregnancy	6/8
Malary, 2021 [26]	Iran	Cross-sectional study	To identify the factors contributing to female low sexual desire and sexual distress during pregnancy	295 pregnant women	Sexual interest and desire inventory-female and female sexual distress scale-revised	Low sexual desire and sexual distress are relatively common sexual experiences during pregnancy. the trimester of pregnancy has a notable influence on sexual distress in women due to the psychological and physiological changes in each trimester. That is why, as pregnancy progresses, all sexual functions decrease. However, higher sexual desire scores are observed in the second trimester. Likewise, satisfaction with sexual foreplay positively influences sexual response during pregnancy	7/8
Sim, 2020 [27]	USA	Cross-sectional study	To examine the pooled associations between testosterone and sexual desire in the couple during pregnancy	58 individuals (29 heterosexual couples) expecting their first child across the prenatal period	Changes in testosterone and two forms of sexual desire (solitary, dyadic)	The pregnant couple adopts the role of parenthood, focusing more on parenting over mating. This new role is marked by changes in testosterone levels. During pregnancy, an increase in testosterone levels is observed in the pregnant woman and a decrease in the man, and these changes in serum testosterone levels are associated with lower levels of dyadic desire in both partners. However, testosterone levels do not influence sexual desire alone	8/8
Jamali, 2018 [28]	Iran	Cross-sectional study	To evaluate men’s sexuality and their attitude toward sexual relationship during their wives’ pregnancy	272 husbands of pregnant women	International Index of Erectile Function Questionnaire	Men usually have a negative attitude towards sex during pregnancy, which affects the pregnant partner’s sexual behaviour. The most prevalent sexual dysfunction was desire disorder and intercourse Dissatisfaction, being more prevalent in the third trimester, where the sexual relationship of many couples become limited or stops altogether. This is due to concerns over labour and the foetus health, and discomfort about the distension of the woman’s stomach intensify	7/8
						Most women, as a result of their physiological changes experienced in the second trimester of pregnancy, find sex more comfortable in this period and resume their sexual relationship, which can affect their husband’s sexual behaviours and lead to have a better sexual performance at this time for them	
Fernández-Carrasco, 2020 [19]	Spain	Cross-sectional study	To see how sexual desire behaves during pregnancy in both partners	108 women and 108 men (n = 216)	Spector et al. sexual desire inventory	Sexual desire decreases during pregnancy in both men and women, the latter being the one with lowest levels of sexual desire, in terms of both solitary and dyadic desire. In the first trimester, pregnant women have the lowest levels of sexual desire, while it is in the third trimester that men have the lowest levels, which may be related to the physiological changes that occur in women, as the woman may be regarded as a mother instead as the object of sexual desire and for fear of harming the fetus as a result of sexual encounter. In contrast, in this third trimester, men’s levels of solitary desire increase	6/8
Erbil, 2018 [29]	Turkey	Cross-sectional study	To examine sexual function of pregnant women in the third trimester of pregnancy	125 healthy and married pregnant women in the third trimester of pregnancy	Female Sexual Function Index (FSFI)	Sexual function of pregnant women in the third trimester were negatively affected, having the lowest scores in the second half (weeks 32–40)	6/8
Güleroğlu, 2014 [1]	Greece	Cross-sectional study	To evaluate sexual functions of the pregnant women and to determine the factors that negatively affect their sexual health	306 pregnant women	Female Sexual Function Index (FSFI)	Most pregnant women have sexual response disorders. Sexual desire and arousal disorders are the most prevalent. The characteristics of the pregnant woman (age, romantic situation, education) and of the pregnancy (pain, bleeding, trimester of pregnancy) negatively affect the sexual response	6/8
Gamusay, 2021 [30]	Turkey	Cross-sectional study	To investigate body image and sexual function of Turkish pregnant women and their partners	254 pregnant women and their 254 partners	Personal Information Form, Body Image Scale, FSFI, and Arizona Sexuality Experiences Scale-Male Form	In the third trimester of pregnancy, couples experience more sexual dysfunction than in the other two trimesters. This may be related to the pregnant woman’s body image	7/8
Balestena-Sánchez, 2014 [31]	Cuba	Cross-sectional study	To determine the influence of pregnancy in the woman’s sexuality	147 pregnant women	Frequency of coitus per week	A woman’s sexuality changes during pregnancy. The second trimester is the most positive, as the number of coitus and levels of excitement and satisfaction are higher than during the rest of the pregnancy. This is because pregnancy is a time of physical, psychological, and spiritual changes. Therefore, pregnant women who have fewer physical symptoms have better sexuality	6/8
Soares, 2020 [32]	Brazil	Cross-sectional study	To assess the sexual function of pregnant women and the influence of sociodemographic, obstetric, and behavioural factors on sexual dysfunction	261 pregnant women	Sociodemographic, obstetric, and behavioural variables, and FSFI.	Sexuality during pregnancy is influenced by many factors including physical, psychological, cultural, social, and religious	6/8
						During pregnancy, beliefs about sexuality may affect the couple negatively and even cause conflict. It is therefore important to understand their specificities in order to cope with the problems inherent to pregnancy. In addition, this study shows that demographic factors and the way in which women are cared for during pregnancy (in public or private settings) have a significant influence on sexuality. However, it also shows that parity is directly related to sexual lubrication and satisfaction	
Bataglia-Doldan, 2014 [33]	Paraguay	Cross-sectional study	To describe the changes observed in women’s sexual activity during pregnancy	321 pregnant women	Interviews. Parenthood and demographic data	In 80% of pregnancies, sexual behaviour is modified. Sexual desire and satisfaction decrease in the first and third trimesters, while an increase is seen in the second trimester. In this study it can be seen that education plays a fundamental role, since information during pregnancy on issues such as sexuality leads to reduced sexual dysfunction	6/8
Alidost, 2021 [34]	Iran	Cross-sectional study	To examine the relationship between the wealth index and pregnancy-related anxiety in each trimester of pregnancy and their effect on sexual dysfunction	450 pregnant women	FSFI, Pregnancy-Related Anxiety Questionnaire, and Wealth Index	Pregnancy is associated with specific tensions and concerns about the new stage, which cause anxiety. a higher mean score of pregnancy-related anxiety is found in the third trimester, as concerns are focused on the state of the foetus and not on the pregnant woman, as in the previous two trimesters. This causes a decrease in sexual activity in the third trimester for fear of harming the foetus. In addition, in the first trimester, physical discomfort and emotional changes also lead to a decrease in sexuality. This is why the second trimester is the one with the least changes in terms of sexuality	6/8
						This anxiety increases due to different factors. It is directly proportional to age and indirectly to educational level. All this indicates that a pregnant woman who has resources and, therefore, a good wealth index to solve her doubts and fears about pregnancy will experience lower levels of sexual dysfunction during this stage	
Aydin, 2015 [35]	Turkey	Controlled cross-sectional study	To compare sexual functions of pregnant and non-pregnant women	246 pregnant women	FSFI.	When comparing the sexual functions of pregnant and non-pregnant women, a higher sexual dysfunction was observed in the first group (90% compared to 60% in the other group)	6/8
						The physiological and psychological changes a woman experiences during pregnancy have an impact on her sexual functions	
						In the first trimester, physical discomfort and psychological adaptations have an impact on decreasing sexual activity and sexual desire	
						In the second trimester, sexual desire increases as there is a decrease in symptoms such as nausea and vomiting	
						In the third trimester, sexual function decreases due to physical discomfort and fear of harming the foetus	
						In addition, it is argued that the economic situation has an impact on sexuality during pregnancy, directly affecting it	
						In conclusion, couples need to be counselled regarding the impact of pregnancy on sexual functions	
Angin, 2020 [36]	Turkey	Cross-sectional study	To compare the female sexual function index and sexual function of their partners between groups of pregnant and non-pregnant Turkish women	252 healthy pregnant and 69 healthy non-pregnant women	FSFI and ARIZONA scores of their partners	Sexual function decreases during pregnancy in both members of the couple. This process is influenced by many factors such as socio-cultural factors, age, etc. one of these factors is overweight; thus, overweight women in the third trimester of pregnancy had weaker sexual functions compared to normal-weight women. It is important for health professionals to warn and educate pregnant couples about sexuality during pregnancy in order to decrease sexual dysfunction	6/8
Gerges, 2022 [37]	Lebanon	Cross-sectional study	To compare women’s perceptions of their sexual functions before and during pregnancy, and to investigate correlates of sexual dysfunction in pregnant women	433 pregnant women	Pregnancy Sexual Response Inventory	Sexuality during pregnancy is affected by many factors, including anxiety and eating disorders. This study reveals the need to inform couples during pregnancy about the constant change in the pregnant woman’s body and the relationship between body image and sexuality. In this way, the couple will obtain information about sexuality during pregnancy so that anxiety levels will decrease and sexual dysfunctions during pregnancy will be avoided	7/8
Babazadeh, 2012 [38]	Iran	Qualitative descriptive study	To evaluate Iranian women’s perception of sexual activity during pregnancy	33 pregnant women	4 semi-structured group interviews	Most women reported a decrease in sexual desire and frequency of intercourse during pregnancy, as well as sexual satisfaction. However, in some cases, decreased sexual desire and activity does not lead to changes in satisfaction, which remains full when sexual activity occurs	9/10
						Moreover, in many cases, these changes in sexuality affect the couple’s relationship, which in some cases deteriorates to the point of divorce or infidelity on the part of the husband who seeks to satisfy his sexual needs	
Yanikkerem, 2016 [39]	Turkey	Cross-sectional study	To evaluate pregnant women’s sexual function and marital adjustment	298 pregnant women	Golombok Rust Inventory of Sexual Satisfaction Scale and Marital Adjustment Scale	Sexual activity during pregnancy decreases, with the third trimester having the lowest levels. Fear of harming the foetus or the pregnant woman is one of the main reasons why coital frequency decreases. In addition, abdominal distention makes intercourse difficult	7/8
						Sexuality is a Taboo topic in Turkey and that is why pregnant couples are not informed of these normal changes that occur during pregnancy, and why they assume many myths about sex during pregnancy to be true. This is why it is very important for healthcare personnel to advise and inform couples so that sexual dysfunctions during this period can be decreased	

FSFI, Female Sexual Function Index.

### Sexual Desire and Pregnancy

Gestation is a period marked by different factors, including physiological and psychological ones [32], which affect women to a greater extent, but also men [19].

On the one hand, the pregnant partner assumes the role of motherhood/parenthood [23–25] and readjusts their sexual and cohabitation expectations (*p* < 0.01). This poses a new sexual response that must be readjusted as the pregnancy progresses and the circumstances of the pregnancy change.

Expectant parents need to be informed by healthcare professionals about the changes that occur in this regard and how they can adapt to them [33–36]. This would decrease the prevalence of sexual dysfunction during pregnancy by 80%–90% [1, 33, 34], as anxiety [34] and fear related to misinformation in this area would be directly associated with sexual distress [26] and, thus, sexual dysfunction (*p* < 0.01).

### Changes in Women’s Sexual Desire During Each Trimester of Pregnancy

For women, the first trimester of pregnancy is generally marked by symptoms such as sickness or nausea, which prevent them from feeling well enough to engage in sexual intercourse. Thus, both solitary and dyadic sexual desire decrease, resulting in reduced frequency of sexual intercourse [24].

In the second trimester of pregnancy, levels of both solitary and dyadic sexual desire increase to levels similar to pre-pregnancy levels (*p* < 0.02) [25, 26, 28]. This is favoured by decreased discomfort and emotional changes associated with pregnancy [24, 25].

In the third trimester of pregnancy, levels of both sexual desire and sexual satisfaction are lower (*p* = 0.004). In the last weeks of gestation [27–33], abdominal distension in the pregnant woman and concern for the baby’s wellbeing often lead to the cessation of sexual intercourse (*p* < 0.01) [28].

### Modification of Men’s Dyadic Sexual Desire in Each Trimester of Pregnancy

As with women, psychological changes and concerns arise in men, which affect their sexual response during this new stage. Men’s sexual attitude during pregnancy is often negative [28, 38]. Fear of harm to the pregnant woman or foetus results in a significant reduction in frequency of sexual relations [24].

Another important factor affecting men’s sexual desire is the change of roles within the couple. Some men report that they see their partner more as a mother than as a sexual partner [19, 25, 39].

In the second trimester, the limitations are lower, which results in a positive attitude on the part of the woman. This positively affects the man’s sexual desire, which increases [24, 33]. The fact that women demand more sexual attention from their partners increases sexual desire in men [19].

In the third trimester, the new body image of women [30] and the difficulty in having sex with a protruding abdomen [30] result in a decrease in the frequency of sexual intercourse, even stopping altogether in many cases (Chi squared = 38.420; *p* = 0.000). In this trimester, and for the same reason (weight gain and increase in abdominal size), men may find their partners less attractive, and this may cause a decrease in dyadic sexual desire at the expense of an increase in solitary sexual desire [19, 37].

### Similarities and Differences Between Male and Female Sexual Desire Throughout Pregnancy

Though not in the same way or at the same time, changes occur during pregnancy that affect the sexual desire of both partners. For instance, serum testosterone levels decrease in both partners, which affects dyadic sexual desire (*p* < 0.001; 95% CI [−0.03, −0.01]) [27]. In this regard, women have the lowest levels of dyadic sexual desire, particularly in the first trimester. In contrast, men have the lowest levels of dyadic sexual desire in the third trimester [19].

Levels of solitary sexual desire in women drop sharply during the first trimester of pregnancy, increase slightly in the second trimester, and continue to increase in the third trimester, but without reaching pre-gestational levels. In men, however, although solitary sexual desire also decreases, it does so only slightly in comparison with their partner [19].

## Discussion

Among the most relevant findings of this review, it is noteworthy that women reported a decrease in sexual desire during pregnancy, both in terms of dyadic and solitary desire. This was attributed to different particularities, some of them related to the pregnant woman, such as her sentimental situation or educational level, and others related to the gestation, such as the trimester or the appearance of bleeding or pain, and all of them evidenced a negative influence on sexual response [1, 19, 33]. These findings are consistent with other studies that report a decrease in frequency of sexual activity during pregnancy (intercourse and orgasm, among others), although other research found that some women experienced an increase in sexual desire during pregnancy, owing to emotional and physiological reasons [40].

Conversely, changes during pregnancy can have a negative influence on the sexual activity of both the pregnant woman and her partner [41], even causing sexual dysfunctions such as dyspareunia, inhibition of sexual desire, and erectile or orgasmic difficulties. These dysfunctions are usually transitory, but when the problem is not timely identified and treated, such dysfunctions may be persistent and even extend beyond the end of pregnancy [42–44]. Changes are observed in the sexual response of both partners, as they readjust their sexual priorities and cohabitation expectations and prepare for a new stage where the role of parent takes on utmost importance [24]. However, it has been found that during the third trimester, men’s sexual desire for solitary sex increases [19].

How age affects sexuality during pregnancy is a matter of debate, as different studies show differing results. Some studies indicate higher levels of sexual desire and greater coital frequency in young pregnant women (<35 years) [45, 46], in line with the results obtained in the present study, which indicate that with increasing age, the prevalence of sexual dysfunction also increases, both in women and their partners [34]. In contrast, other authors find no association between the age and sexuality during pregnancy variables [47]. The same is true for parity; some authors describe decreased coital frequency throughout pregnancy in multiparous women [48, 49], while other studies have found this decline to be typical of nulliparous pregnant women [50]. The selected results show that nulliparous women had significantly lower means for lubrication and sexual satisfaction. This may be due to the fact that nulliparous women may be more vulnerable to emotional factors due to lack of experience and to fears and anxieties related to the first pregnancy [32].

Education plays a key role in this issue, as the knowledge acquired during pregnancy related to sexuality and its components leads to reduced sexual dysfunction [33]; these results are in line with other findings that reveal that educational level plays an important role in sexual behaviour during pregnancy [51]. Lack of information, especially in nulliparous pregnant women, often inhibits sexual practices due to fear of complications, premature birth, or miscarriage [52]. In addition, cultural and religious beliefs also affect sexuality during pregnancy [53]. The study by Sapién and Córdoba [44] proves that men’s beliefs that sex during pregnancy can be harmful to the foetus affect sexual relations with their pregnant partners. This particularly affects vaginal intercourse, the frequency of which decreases as pregnancy progresses and may even be suppressed as soon as the pregnancy is medically diagnosed [40], findings that fully coincide with the ones found in the present study. Many couples become very fearful of causing any kind of harm to the pregnant woman or the foetus, so they decrease coital frequency [24] or in some cases, they may even completely interrupt sexual relations. This can be due to concerns about childbirth [28] or because the man regards his partner as a mother rather than as a sexual partner [19].

A relationship exists between sexual desire and the marital status of the pregnant woman. Marital status has a decisive influence on the desire for pregnancy; women who are married have greater willingness to become pregnant than unmarried women. There is a statistically significant relationship between the desire for pregnancy and the pregnant woman’s perception of sexual desire for her husband. This is why most married women, during pregnancy, continue to find their partners sexually desirable, and in turn their sexual desire for their partner is greater [54]. However, the results found in the present research indicated that arranged marriages and marriages of more than 10 years had a negative effect on the sexual activity of pregnant women [1].

Concerning psychological aspects, pregnancy is a new stage in a couple’s life that involves readaptation, for which communication is fundamental. It is therefore important to examine how the pregnant woman and her partner perceive physiological changes as, in some cases, her new physical image can make the pregnant woman feel insecure or less attractive [41]. During pregnancy, it has also been observed a state marked by anxiety, fear and concern about what is or is not correct in terms of sexual behaviour. This leads to attitudes that are influenced by fear of obstetric and gynaecological complications and which are often misunderstood by the partner. In addition, beliefs and myths about harming the foetus lead many couples to decrease or suppress sexual relations [55], as discussed above. The results found in the present study reinforce how pregnancy is, in many cases, marked by sexual distress stemming from the psychological changes experienced by both partners [26]. Women’s body image differs by trimester and the perception of this construct became more negative in the third trimester. A positive body image positively influenced the sexual behaviour of the partners, and a significant positive correlation was found between body image and sexual behaviour in pregnant women [30].

Finally, many studies warn of the need for healthcare professionals to provide sexuality education to expectant parents [33, 34], especially the healthcare workers involved in pregnancy control (obstetricians, midwives and nurses) having a good opportunity to educate couples during childbirth preparation courses, as well as in successive follow-up consultations throughout the pregnancy. As Brtnicka et al. [56] determined, sexual dysfunctions may exist during pregnancy, and these are often guided by fear of harming the foetus. Men also reported fear of hurting women during pregnancy, and women are afraid of insufficient satisfaction from their partner. To prevent sexual dysfunctions, which may even persist after pregnancy, it is important for the couple to readjust their sexuality. In other words, myths and fears must be eradicated, new positions and sexual games must be adopted, and even sexual relations in which penetration does not exist or is not the central axis, so that sex during pregnancy can be satisfactory for both partners [24, 26]. These findings concur with others that point to the lack of information many couples have about their sexuality and the changes that occur during pregnancy [25].

This systematic review has a number of limitations. Firstly, there are not many studies on sexual desire (dyadic and solitary) during pregnancy in the last 10 years. In addition, studies on male sexuality during this stage are also very scarce. For this reason, the literature found was limited, which poses the need to carry out further research on this topic in the future. Besides, although heterosexual couples are the most common, there are also other homosexual family models or even those with different sexual orientations, so it would be interesting to know in future investigations how sexual desire during pregnancy affects these types of family models, currently considered a minority. Likewise, this review has been carried out on studies in several countries and continents, so it would be equally interesting to investigate each specific context to identify the religious or cultural differences that can affect the sexual behavior of the couple at a global level. The language in which the search was carried out (English and Spanish) can also be established as a limitation, although we are aware that most scientific publications are made in English, since it is possible that some relevant work published in any other language has not been selected. At the same time, it is worth mentioning within the limitations that the period of time where the search was framed was the last 10 years, with the possibility that some work that was previously published may have been lost.

### Conclusion

Sexual desire changes during pregnancy in both the pregnant woman and her partner, decreasing for couples in the first trimester due to the physical discomfort of pregnancy as well as the psychological changes experienced by each partner in the face of assuming the new role of parents. Conversely, desire increases in the second trimester, motivated by an improvement in the physical discomfort typical of early pregnancy and emotional stabilisation, only to decline again in the third trimester. This reduced desire may be due to various reasons, such as the father’s fear of harming the foetus or his partner, or the difficulty in having sexual relations when there is abdominal distension, which leads to a decrease in the coital frequency and, in many cases, to the complete interruption of sexual intercourse.

Healthcare professionals should have a clear understanding of how pregnancy affects the sexual desire of both men and women, as they should provide objective information and clarify any doubts about sexuality during pregnancy. Therefore, pregnancy control visits and childbirth preparation courses allow healthcare professionals involved in pregnancy care to interact with the couple, to advise them (so that they can experience a satisfactory sexuality), and to prevent the appearance of possible sexual dysfunctions due to the changes brought about by pregnancy or to explore interventions and strategies to address it.
